# Comparison of Macrophage Immune Responses and Metabolic Reprogramming in Smooth and Rough Variant Infections of *Mycobacterium mucogenicum*

**DOI:** 10.3390/ijms23052488

**Published:** 2022-02-24

**Authors:** Minji Kang, Ho Won Kim, A-Reum Yu, Jeong Seong Yang, Seung Heon Lee, Ji Won Lee, Hoe Sun Yoon, Byung Soo Lee, Hwan-Woo Park, Sung Ki Lee, Seungwan Lee, Jake Whang, Jong-Seok Kim

**Affiliations:** 1 Department of Medical Science, Chungnam National University, Daejeon 35365, Korea; mjkangs92@naver.com; 2 Myunggok Medical Research Institute, College of Medicine, Konyang University, Daejeon 35365, Korea; kimong104@naver.com (H.W.K.); kyoaor22@hanmail.net (A.-R.Y.); smileday0103@naver.com (J.W.L.); cordelia_sun@naver.com (H.S.Y.); 3 Korea Mycobacterium Resource Center (KMRC), Department of Research and Development, The Korean Institute of Tuberculosis, Osong 28158, Korea; nonr@daum.net (J.S.Y.); seung6992@hanmail.net (S.H.L.); 4 Department of Ophthalmology, Konyang University Hospital and College of Medicine, Daejeon 35365, Korea; kannylee@naver.com; 5 Department of Cell Biology, Konyang University College of Medicine, Daejeon 35365, Korea; hwanwoopark@konyang.ac.kr; 6 Department of Obstetrics and Gynecology, Konyang University Hospital, Daejeon 35365, Korea; sklee@kyuh.ac.kr; 7 Department of Medical Science, Konyang University, 158 Gwanjeodong-ro, Daejeon 35365, Korea; slee1@konyang.ac.kr

**Keywords:** *Mycobacterium mucogenicum*, immune response, metabolism, TLR2, glycolysis, mitochondrial respiration

## Abstract

*Mycobacterium mucogenicum* (*Mmuc*), a rapidly growing nontuberculous mycobacterium (NTM), can infect humans (posttraumatic wound infections and catheter-related sepsis). Similar to other NTM species, *Mmuc* exhibits colony morphologies of rough (*Mmuc*-R) and smooth (*Mmuc*-S) types. Although there are several case reports on *Mmuc* infection, no experimental evidence supports that the R-type is more virulent. In addition, the immune response and metabolic reprogramming of *Mmuc* have not been studied on the basis of morphological characteristics. Thus, a standard ATCC *Mmuc* strain and two clinical strains were analyzed, and macrophages were generated from mouse bone marrow. Cytokines and cell death were measured by ELISA and FACS, respectively. Mitochondrial respiration and glycolytic changes were measured by XF seahorse. Higher numbers of intracellular bacteria were found in *Mmuc*-R-infected macrophages than in *Mmuc*-S-infected macrophages. Additionally, *Mmuc*-R induced higher levels of the cytokines TNF-α, IL-6, IL-12p40, and IL-10 and induced more BMDM necrotic death. Furthermore, our metabolic data showed marked glycolytic and respiratory differences between the control and each type of *Mmuc* infection, and changes in these parameters significantly promoted glucose metabolism, extracellular acidification, and oxygen consumption in BMDMs. In conclusion, at least in the strains we tested, *Mmuc*-R is more virulent, induces a stronger immune response, and shifts bioenergetic metabolism more extensively than the S-type. This study is the first to report differential immune responses and metabolic reprogramming after *Mmuc* infection and might provide a fundamental basis for additional studies on *Mmuc* pathogenesis.

## 1. Introduction

Nontuberculous mycobacteria (NTMs) are common in nature and exist in water sources, soil, and vegetation as environmental organisms. Currently, more than 200 species are recognized as NTMs, and new species are identified every year [1,2]. Although relatively few species of NTMs cause disease in human, the incidence of human NTM infection appears to have increased significantly in recent decades due to causes such as an aging population, immunosuppression, and broad-spectrum antibiotic use [1,2,3]. In addition, most NTMs that cause infections are challenging to treat and worsen the patient’s prognosis because they are resistant to the first-line drug for tuberculosis [4]. According to their growth rate, NTMs that grow on the media within or after 7 days are defined as rapidly growing mycobacteria (RGM) and slowly growing mycobacteria (SGM), respectively [5]. Of these, *Mycobacterium mucogenicum* (*Mmuc*) belongs to the RGM group [6].

*Mmuc* has rarely been reported to infect humans, and cases are most commonly seen in immunocompromised patients [6]. Their isolation may be considered clinically important because RGMs are recovered from various environmental sources, including water, and they survive by forming biofilms and by interacting with protozoa [6,7]. After *Mmuc* was first reported as a *Mycobacterium chelonae*-like organism (MCLO) in a peritonitis outbreak in 1982, *Mmuc* strains have been detected in a wide range of infection sites, such as the central nervous system, skin, and lungs [8,9,10]. In particular, the most clinically significant infections are posttraumatic wound infections and catheter-related sepsis [6,9]. In particular, *Mmuc* has been identified as a water contaminant in hospitals. Possible causes of these opportunistic infections include a poorly disinfected organ indwelling catheter and contamination of the water used for surgeries, such as dialysis, and the water used for laboratory testing [8]. Furthermore, *Mmuc* was shown to be more resistant to chlorine than other bacteria, including *M. fortuitum* and *Escherichia coli*, and therefore, it has a higher probability of contamination [3]. Similar to other pathogenic NTMs, *Mmuc* can physically display various colony morphologies, including rough (R) and variable smooth (S) types [11,12]. This colony-based distinction is dependent on the presence or absence of surface-associated glycopeptidolipids (GPLs) [13]. *Mmuc* is classified as the S-type if GPLs are present and as the R-type if GPLs are not [11,13]. The presence or absence of GPLs is related to meaningful pathological aspects such as biofilm formation or sliding motility, interaction with host cells, intracellular trafficking in macrophages, and virulence, which affects the clinical severity of the infection [11]. In general, the R-type of *Mycobacterium* spp. causes a more severe inflammatory response in macrophages or mice than the S-type [13,14]. The morphological classification of *Mmuc* and patient case reports have been studied [12], but no studies have assessed immunological differences based on morphological analyses.

In the present study, the immune responses to the standard ATCC49650 strain (*Mmuc*-ATCC) and the R-type (*Mmuc*-R; KMRC 00136-76003) and S-type (*Mmuc*-S; KMRC 00136-76002) clinical strains were first compared. Here, we examined immune responses based on the fact that virulent mycobacteria can survive and replicate within macrophages as intracellular bacteria. Additionally, we examined the steps of apoptotic cell death because it is regarded as an innate intracellular response designed to limit the multiplication of intracellular pathogens. In addition, we attempted to compare and analyze the cytokine patterns of each strain according to the activation of macrophages. Finally, extracellular flux analysis was performed to assess the modulation of the energy metabolism of BMDMs infected with *Mmuc*-ATCC, *Mmuc*-S, and *Mmuc*-R.

## 2. Results

### 2.1. Colony Morphology, Scanning Electron Microscope, and Thin Layer Chromatography

**Figure 1:**
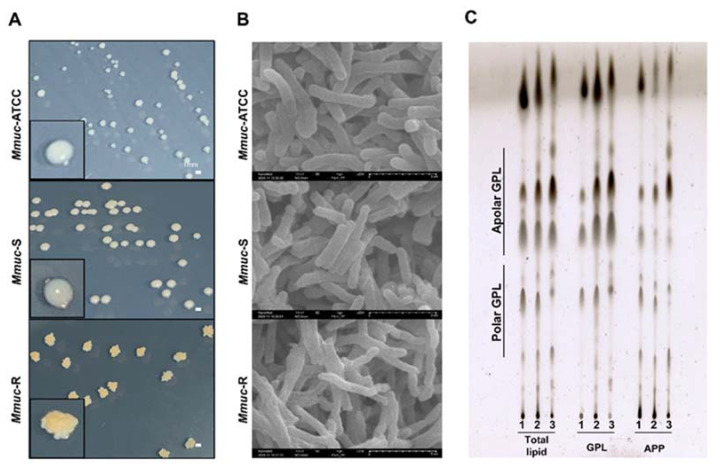
*M. mucogenicum* morphologies. (**A**) Colony characteristics of *M. mucogenicum* in 7H10 media after 7 days. (**B**) Scanning electron microscopy images of the *Mmuc*-ATCC (ATCC 49650) strain and other clinical strains. (**C**) The purified GPLs and the acetone-precipitated pellet (APP) from total lipids extracted from *Mmuc*-ATCC (lane l), *Mmuc*-S (lane 2), and *Mmuc*-R (lane 3) were analyzed by thin-layer chromatography (TLC) using chloroform/methanol (9:1, *v*/*v*) as the mobile phase. GPL: glycopeptidolipids.

One physical characteristic of *Mmuc*, similar to other NTMs, is the ability to exhibit both rough and smooth colony morphologies on 7H10 agar (Figure 1A). The reference strain *Mmuc*-ATCC49650 and clinical strain KMRC 00136-76002 showed smooth colonies, and the clinical strain KMRC 00136-76003 showed rough colonies. The S-type colonies exhibited circular, convex, and marginal forms with a slightly translucent border. Additionally, the S-type colonies were creamy-white and small in size. Unlike the S-type colonies, the R-type colonies exhibited irregular forms and dry surfaces with many wrinkles and crests. Additionally, the R-type colonies were more yellowish and small in size. Scanning electron microscope (SEM) was used to characterize the surfaces of *Mmuc* strains in more detail (Figure 1B). The SEM results once again confirmed that the three strains were rod-shaped and that both S-types of *Mmuc*, ATCC49650 and KMRC 00136-76002, showed regular surfaces, while the R-type of *Mmuc*, KMRC 00136-76003, had irregular and rough surfaces and was relatively cohesive. As expected from the thin layer chromatography (TLC) data, lipid differences were observed between the S- and R-types. The S-type of *Mmuc* appeared darker in the polar GPL region than the R-type (Figure 1C). For this reason, the *Mmuc* reference strain will hereafter be abbreviated as *Mmuc*-ATCC, the S-type clinical strain (KMRC 00136-76002) will be abbreviated as *Mmuc*-S, and the R-type clinical strain (KMRC 00136-76003) will be abbreviated as *Mmuc*-R.

### 2.2. Intracellular Growth Pattern of M. mucogenicum in Macrophages

**Figure 2:**
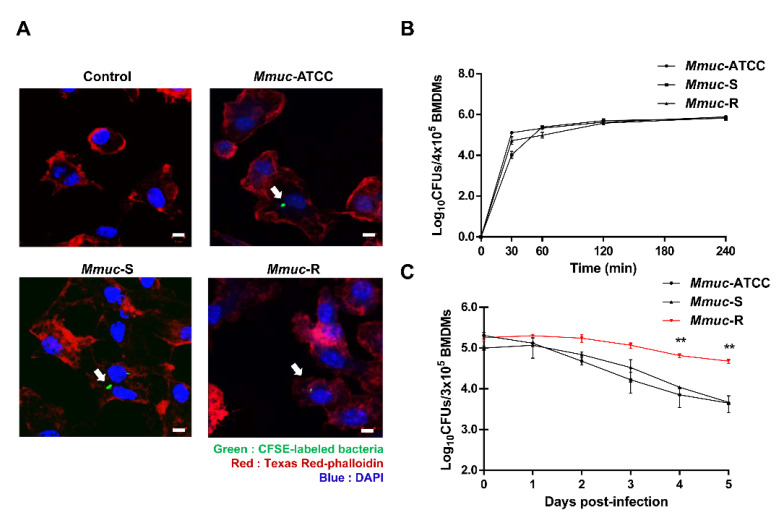
*M. mucogenicum* is an intracellular bacteria. (**A**) Intracellular staining of CFSE-coupled *Mmuc* in BMDMs infected at an MOI of 10 bacteria per cell for 4 h. Cortical F-actin was stained using phalloidin-Texas red, and nuclei were stained with DAPI. Scale bar = 5 μm. (**B**) Infection kinetics of *M. mucogenicum* in BMDMs during the first 4 h postinfection. BMDMs were infected with *Mmuc* at an MOI of 10. At each indicated time point, cells were washed three times and lysed with 0.05% Triton X-100 containing distilled water to release intracellular bacteria. This experiment was repeated three times with similar results. (**C**) Growth profiles of *Mmuc* within BMDMs over a 5-day period after infection. BMDMs were infected at an MOI of 10. Noninternalized bacteria were washed off after 4 h. The number of bacterial colony-forming units (CFUs) was determined at the indicated time point after removing the supernatant. Values are the means of triplicate samples, and error bars represent standard deviations (SD) (** *p* < 0.01). This experiment was repeated three times with similar results.

Since *Mmuc* was first discovered in 1995, most research has been clinical in nature, and thus, it is unknown whether it infects macrophages in vitro. Therefore, in this study, we first tried to confirm whether *Mmuc* was capable of infecting macrophages. To do this, each type of *Mmuc* was labeled with CSFE and then used to infect mouse BMDMs at an MOI of 10 bacterium per cell. Phagocytosis of *Mmuc*-ATCC, *Mmuc*-S, and *Mmuc*-R into macrophages occurred at similar levels (Figure 2A). We further investigated any differences from immediately after to 4 h after infection, but no differences were observed for any of the strains (Figure 2B).

The ability to replicate and survive within a macrophage is vital for intracellular pathogen virulence [15]. Therefore, we tried to assess differences in replication and survival in host cells depending on the morphological differences of *Mmuc*. As shown in Figure 2C, the bacterial numbers of *Mmuc*-ATCC and *Mmuc*-S gradually decreased until 5 days after BMDM infection, whereas the number of *Mmuc*-R did not decrease. These results indicated that while the levels of S- and R-type *Mmuc* phagocytosis into macrophages were similar, the intracellular and multiplication rates of the R-type *Mmuc* was significantly higher than the investigated S-type strains. Therefore, we hypothesized that the R-type of *Mmuc* is more virulent than the S-type of *Mmuc* due to its ability to survive and multiply inside macrophages.

### 2.3. M. mucogenicum Infection Induces BMDM Death

**Figure 3:**
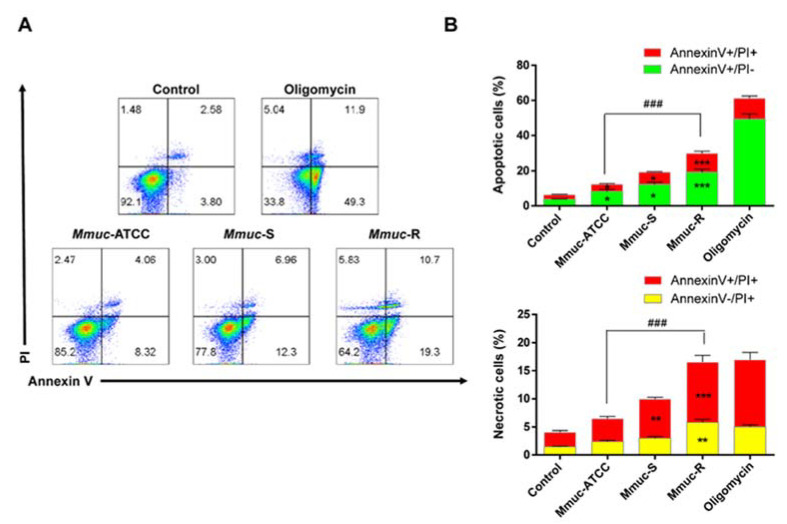
Quantitative analyses of the necrosis and apoptosis of BMDMs infected with *M. mucogenicum*. Representative results of Annexin V–PI staining (**A**) and quantitative analysis (**B**). BMDMs were infected with *Mmuc* at an MOI of 10 for 24 h. After incubation, cells were collected, and FITC-Annexin V and PI were added. Samples were analyzed by flow cytometry. Values are mean ± SD of three samples (* *p* < 0.05, ** *p* < 0.01, *** *p* < 0.001 versus control group; ^###^ *p* < 0.001 versus *Mmuc-ATCC* group).

Macrophage cell death can be induced by *Mycobacterium* spp. infection [16]. To determine whether the cytotoxic effects of the *Mmuc*-R and S-type *Mmuc*-ATCC and *Mmuc*-S strains differed, cell death was measured using an Annexin V/PI assay. Exposure to the appropriate fluorescent dye, Annexin V for apoptosis and PI for loss of cell membrane integrity, was used to distinguish between viable, apoptotic, and early and late necrotic cells. When *Mmuc*-ATCC and *Mmuc*-S were infected at an MOI of 10, the levels of apoptotic (Annexin V+/PI− and Annexin V+/PI+) and necrotic (Annexin−/PI+ and Annexin V+/PI+) macrophage death were significantly increased. Both apoptotic cell death and necrosis were higher after *Mmuc*-R infection than after S-type strain infection (Figure 3A,B). Even for the S-type strains, the clinical strain caused more cell death than the standard strain. Overall, the levels of apoptosis, necrosis, and late apoptosis/necrosis were higher in the *Mmuc*-R infection group than in the *Mmuc*-S group. These results indicate that *Mmuc*-R is more cytotoxic to macrophages than S-type *Mmuc*.

### 2.4. M. mucogenicum Induces High Levels of Cytokine Production and Leads to the Phosphorylation of MAPKs in BMDMs

**Figure 4:**
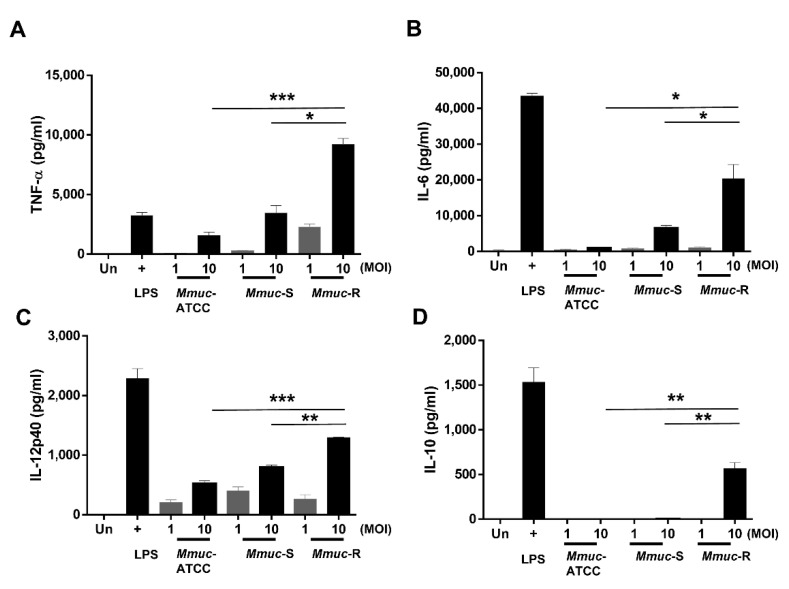
Comparison of inflammatory cytokine production by BMDMs infected with *M. mucogenicum* strains. BMDMs were infected with *Mmuc* strains at an MOI of 1 or 10 for 24 h. The results are presented as the mean pg/mL ± SD for all the experiments performed. Supernatants were collected, and the levels of TNF-α (**A**), IL-12p40 (**B**), IL-6 (**C**), and IL-10 (**D**) were determined by the enzyme-linked immunosorbent assay. Values are mean ± SD of three samples (* *p* < 0.05, ** *p* < 0.01, or *** *p* < 0.001). Un: uninfected.

An excessive secretion of inflammatory cytokines causes tissue damage but is essential for controlling infection. However, in general, the ability to secrete proinflammatory cytokines and virulence do not always correlate, and in the case of *Mycobacterium tuberculosis*, highly pathogenic bacteria induce more secretion of inflammatory cytokines, including TNF-α [17]. We compared the abilities of *Mmuc*-R and the S-types of *Mmuc*, both *Mmuc*-ATCC and *Mmuc*-S, to stimulate the release of cytokines from BMDMs at an MOI of 1 or 10 at 24 h postinfection. Overall, *Mmuc*-R elicited significantly higher levels of the proinflammatory cytokines TNF-α, IL-12p40, and IL-6 than S-type *Mmuc*-ATCC and *Mmuc*-S in an MOI-dependent manner (Figure 4A–C). In particular, macrophages infected with *Mmuc*-R produced significantly more IL-10 than those infected with the S-types *Mmuc*-ATCC and *Mmuc*-S at an MOI of only 10 (Figure 4D). Overall, the cytokine levels of the clinical strains were higher than that of *Mmuc*-ATCC.

**Figure 5:**
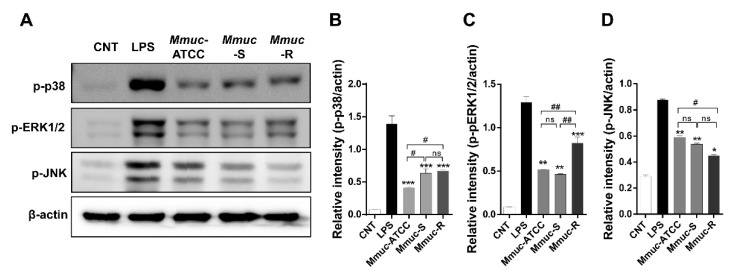
*M. mucogenicum*-induced activation of MAPKs and NF-kB. (**A**) BMDMs were infected with the indicated *Mmuc* strains at an MOI of 10, and protein expression was detected at 30 min. Cell lysates were subjected to SDS–PAGE, and immunoblot analysis was performed using specific antibodies against phospho-p38 (p-p38), phospho-ERK1/2, phospho-JNK, and β-actin. (**B**–**D**) Each protein bands in A were scanned, and relative band intensities were normalized for the β-actin band. The column diagrams represent average relative band intensity with standard error from three independent experiments. * *p* ≤ 0.05, ** *p* ≤ 0.01, *** *p* ≤ 0.001 versus control; ^#^ *p* ≤ 0.05, ^##^ *p* ≤ 0.01 for *Mmuc*-ATCC versus *Mmuc*-S or *Mmuc*-ATCC versus *Mmuc*-R or *Mmuc*-S versus *Mmuc*-R). ns: nonsignificant.

The initial immune response to bacteria, including the interaction between mycobacteria and macrophages, involves the activation of mitogen-activated protein kinases (MAPKs), which play important roles in promoting antimycobacterial activity and the production of proinflammatory cytokines [18]. To investigate the different levels of cytokine production in macrophages and to determine whether cytokine production passes through the MAPK signaling pathway in response to *Mmuc* strains, we analyzed the phosphorylation profiles of p38, ERK1/2, and JNK activation in BMDMs infected with *Mmuc* strains at an MOI of 10 for 30 min. As shown in Figure 5, all *Mmuc* strains induced the phosphorylation of ERK1/2, p38, and JNK at 30 min after stimulation. p38 Phosphorylation was induced to a higher extent in the group infected by the two clinical strains of *Mmuc* than in that infected by the standard strain, and in the case of Erk1/2, *Mmuc*-R induced the highest phosphorylation. JNK levels did not differ in the *Mmuc* strain groups.

### 2.5. M. mucogenicum-Induced Cytokine Production by Macrophages Is Mediated by TLR2 Signaling

**Figure 6:**
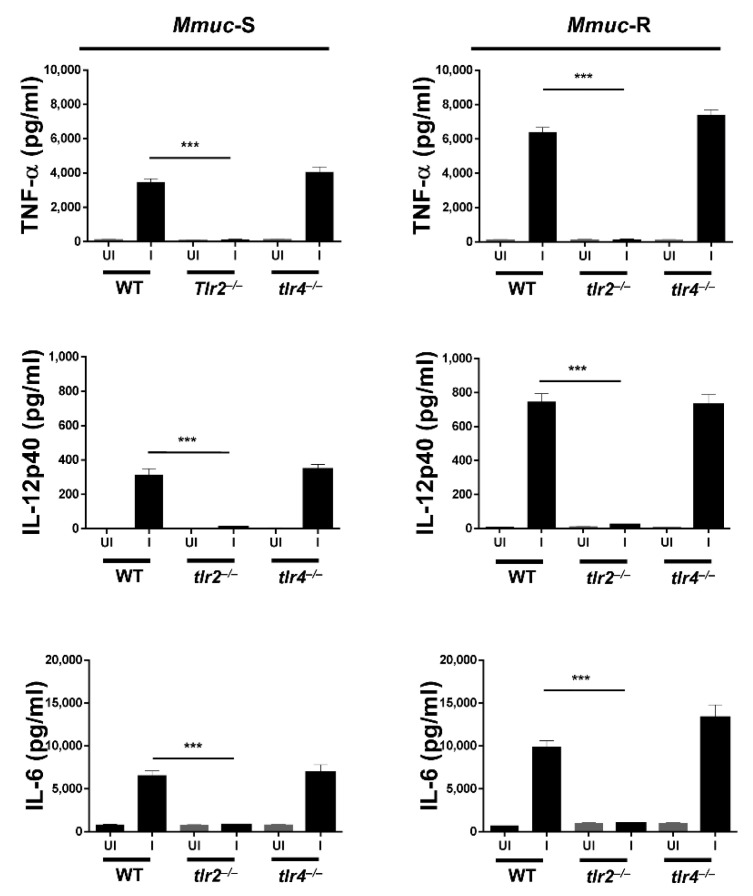
TLR2 plays an essential role in *M. mucogenicum*-induced inflammatory cytokine production by BMDMs. BMDMs from wild-type, TLR2^−/−^, and TLR4^−/−^ mice were infected with *Mmuc* for 24 h at an MOI of 10 and then screened for the secretion of the cytokines TNF-α, IL-12p40, and IL-6 by the enzyme-linked immunosorbent assay. Values are mean ± SD of three samples (*** *p* < 0.001 for WT versus tlr2^−/−^). UI: uninfected, I: infected.

Many *Mycobacterium* spp. are known to recognize Toll-like receptor (TLR) 2 or TLR4 [19]. To identify the cell receptor in response to *Mmuc*, BMDM cultures from TLR2- and TLR4-deficient and wild-type (WT) C57BL/6 mice were established and infected with *Mmuc*-R and *Mmuc*-S at an MOI of 10 (Figure 6). By measuring the levels of proinflammatory cytokines such as TNF-α, IL-12p40, and IL-6 in BMDMs, *Mmuc* was shown to induce an immune response via TLR2. The stimulation of TLR2-deficient macrophages returned the levels of *Mmuc*-induced TNF-α, IL12p40 and IL-6 to the baseline, indicating that TLR2 plays a critical role in *Mmuc*-induced cytokine production in macrophages.

### 2.6. Metabolic Profile of M. mucogenicum-Infected BMDMs

**Figure 7:**
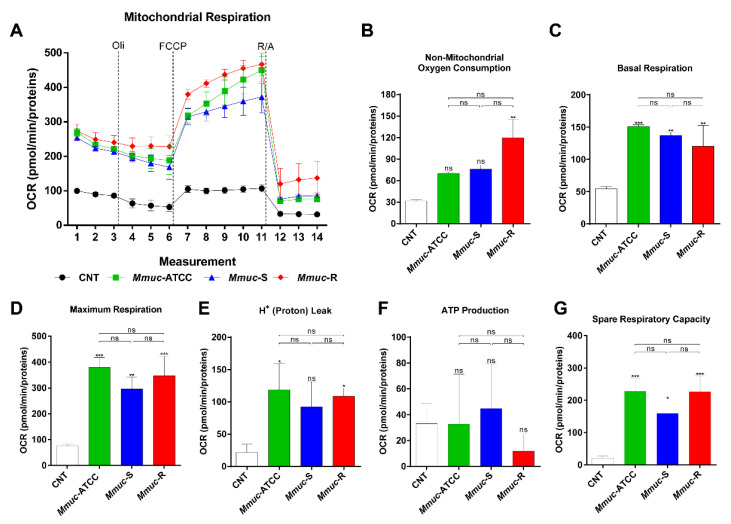
Oxygen consumption rate of *Mmuc*-infected BMDMs. BMDMs were infected with *Mmuc*-ATCC, *Mmuc*-S, or *Mmuc*-R at an MOI of 10 for 24 h. (**A**) Mitochondrial respiration was determined while monitoring oxygen consumption rates (OCRs) with a Seahorse XFp analyzer using a Cell Mito stress test kit. The sequential injection of oligomycin (Oli, 1.0 μM), cyanide-4-[trifluoromethoxy] phenylhydrazone (FCCP, 2.0 μM), and rotenone/antimycin A (R/A, 0.5 μM) is indicated. (**B**–**G**) Representative nonmitochondrial oxygen consumption, basal respiration, maximum respiration, H^+^ (proton) leakage, ATP production, and spare respiratory capacity values were determined using a Seahorse XF Cell Mito stress report generator. The data are normalized to the protein concentration. All data are presented as the mean ± SD (*n* = 3). (* *p* ≤ 0.05, ** *p* ≤ 0.01, *** *p* ≤ 0.001 versus control). ns: nonsignificant.

**Figure 8:**
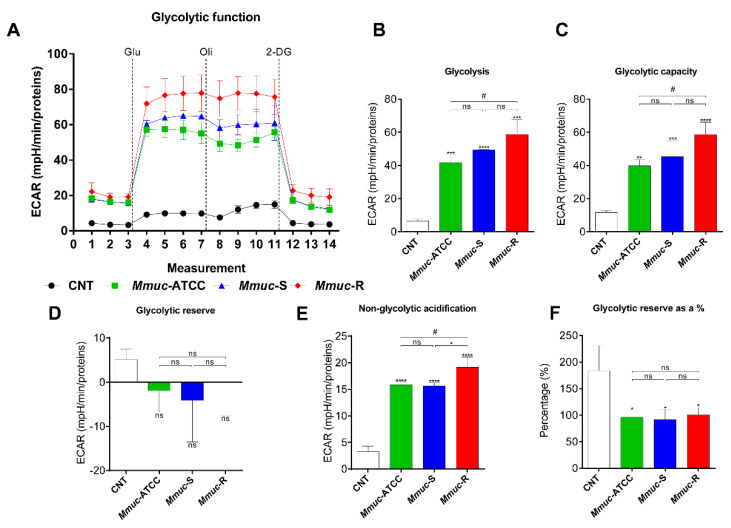
Extracellular acidification profiles and glycolytic parameters of *Mmuc*-infected BMDMs. BMDMs were infected with *Mmuc*-ATCC, *Mmuc*-S, or *Mmuc*-R at an MOI of 10 for 24 h. (**A**) Representative measurements of the extracellular acidification rates (ECARs) in *Mmuc*-infected BMDMs were acquired using the XF Glycolysis Stress Test kit. The sequential injection of glucose (Glu, 10 mM), oligomycin (Oli, 1.0 μM), and 2-deoxyglucose (2-DG, 50 mM) is indicated. (**B**–**F**) Representative glycolysis, glycolytic capacity, glycolytic reserve, nonglycolytic acidification, and glycolytic reserve as a percentage values were determined using the Seahorse XF Cell ECAR report generator. The data are normalized to the protein concentration. All data are presented as the mean ± SD (n = 3). (* *p* ≤ 0.05, ** *p* ≤ 0.01, *** *p* ≤ 0.001, **** *p* ≤ 0.0001 versus control; # *p* ≤ 0.05 for *Mmuc*-ATCC versus *Mmuc*-R). ns: nonsignificant.

Several reports suggest that metabolic alterations of macrophages during infection can also benefit intracellular bacteria [20]. Thus, analyzing how metabolic processes in macrophages are changed during infection can further our understanding of how *Mmuc* causes disease. We examined how the *Mmuc* burden affects oxidative phosphorylation (OXPHOS) and glycolysis in BMDMs (Figure 7 and Figure 8). Figure 7 shows that infection with the *Mmuc*-R and the S-types of *Mmuc*, both *Mmuc*-ATCC and *Mmuc*-S, significantly increased the respiratory parameters: basal respiration, maximum respiration, H^+^ (proton) leakage, and spare respiratory capacity. Only infection with *Mmuc*-R significantly increased the nonmitochondrial respiration parameters (Figure 7B). The basal respiration, maximum respiration, and spare respiratory capacity were significantly increased in all *Mmuc* strain-infected BMDMs (Figure 7C,D,G). Moreover, H^+^ (proton) leakage tended to be increased in all the *Mmuc* strain-infected BMDMs compared to the control (Figure 7E). In contrast, the level of ATP production in *Mmuc*-infected BMDMs was comparable to that in control, but that in the *Mmuc*-R infection group showed a decreasing trend (Figure 7F). Next, we sequentially treated *Mmuc*-infected BMDMs with glucose, oligomycin, and 2-deoxyglucose and measured the glucose metabolism extracellular acidification rate (Figure 8A). The glycolysis, glycolytic capacity, and nonglycolytic acidification parameters were significantly increased in all Mmuc strain-infected BMDMs (Figure 8B,C,E). When infected with *Mmuc*-R, these parameters were significantly increased compared to infection with *Mmuc*-ATCC. In addition, nonglycolytic acidification was significantly higher in cells infected with *Mmuc*-R compared to both S-types. Contrary to the glycolysis, glycolytic capacity, and nonglycolytic acidification parameters, *Mmuc* infection of BMDMs tended to decrease the glycolytic reserve and glycolytic reserve as percentage parameters (Figure 8D,F). In particular, the glycolytic reserve as a percentage parameter was significantly decreased in all *Mmuc* strain-infected BMDMs. In summary, marked glycolytic and respiratory differences were observed between the control and *Mmuc* infection groups, with *Mmuc* infection significantly promoting glucose metabolism extracellular acidification and oxygen consumption in the BMDMs.

## 3. Discussion

Mmuc strains are isolated from water and capable of causing opportunistic infections and can contaminate chemical devices and equipment, which may provide a mechanism for bacteria to enter the bloodstream through wounds. Immunosuppressed patients are at risk of significant morbidity and mortality from exposure to these healthcare environmental pathogens [8]. This study demonstrates for the first time the differences in the innate immune responses, signaling pathways, virulence, and metabolic changes of the S- and R-type clinical strains and the ATCC standard strain of *Mmuc*. The *Mmuc*-R grew as wrinkled and rough colonies on Middlebrook 7H10 agar, whereas *Mmuc*-ATCC and *Mmuc*-S grew into smooth, dome-shaped colonies. In a similar manner, electron microscopy confirmed that the surface of *Mmuc*-R was more wrinkled and rough, while the surfaces of the strains *Mmuc*-ATCC and *Mmuc*-S were less wrinkled. Previous studies on the colony morphotypes of NTMs, including *M. abscessus*, have shown that rough morphotypes lacking GPLs induce a greater inflammatory response and more virulence than smooth morphotypes [5,13].

Our results clearly show that the intracellular survival of *Mmuc*-R within macrophages was higher than that of both S-type strains. According to our data, although *Mmuc*-R had a better chance of surviving in BMDMs than S-type *Mmuc*, whether the bacterial counts of both types of *Mmuc* increased until 5 days after infection could not be determined. In the case of *Mmuc*-R, the number of initially infected bacteria was maintained for 5 days, and the number of S-type strain bacteria decreased severely. Actually, infection of mouse BMDMs with pathogenic *Mycobacterium* spp. does not always increase bacterial numbers. In particular, in the case of *M. abscessus*, depending on the experimental condition or strain type, the number may decrease slightly upon macrophage infection [21,22]. Perhaps this difference is because even if the bacteria were sufficiently multiplied within the cell, they were released to the outside due to cell death and then removed during washing. In addition, although the bacteria are continuously multiplied, the bacterial numbers may nearly appear to be maintained because they are removed by various macrophage suppression mechanisms. Further research should be focused on elucidating the underlying mechanisms in more detail.

Another predictive marker of intracellular pathogen virulence is the confirmation of cytokine secretory capacity. Highly pathogenic bacteria generally induce the secretion of inflammatory cytokines, but they are not always correlated. All strains of *Mmuc* upregulated the proinflammatory cytokines TNF-α, IL-6, and IL-12p40 upon infection, and their levels increased in an MOI-dependent manner. In particular, *Mmuc*-R induced relatively high inflammatory cytokine secretion and was the only investigated strain to induce IL10 production, which is an anti-inflammatory cytokine. Almost all NTMs, including *M. abscessus* and *M. massiliense,* can be divided into rough and smooth types depending on the presence or absence of GPLs, and most R-type strains are highly pathogenic and have high cytokine secretory capacity [23]. In our results, cytokine secretion was higher in the *Mmuc*-R, and the pattern was the same as that for the general NTM strain.

Apoptosis is a programmed cell death mechanism controlled by host balance with a cell signal generated by the cell itself, and necrosis is an unprogrammed cell death caused by external stimuli such as infection. In addition, many reports suggest that necrosis is preferred over apoptosis within macrophages infected by virulent pathogens [16]. In this study, *Mmuc*-R promoted late cell death and a necrotic phenotype of macrophages, suggesting that *Mmuc*-R is more pathogenic during infection and may sufficiently proceed to host cell lysis and tissue injury. Upon comparing the immune responses of *Mmuc* strains, *Mmuc*-R was more virulent than S-type *Mmuc* in all aspects of intracellular viability; the production of cytokines is important for the immune response, induction of cell death, and signaling pathways, similar to the immune response of other RGMs. Furthermore, our study showed that the clinical strains were more virulent than *Mmuc*-ATCC.

Several intracellular bacteria have been reported to shift the bioenergetic metabolism of macrophages via a specific approach that benefits the pathogen [20]. The mechanism by which NTM rewires macrophage energy metabolism to facilitate survival is poorly characterized. Here, we used extracellular flux analysis to explore the modulation of the energy metabolism of BMDMs infected with *Mmuc*-ATCC, *Mmuc*-S, and *Mmuc*-R. *Mmuc* infection induced a metabolic shift of infected BMDMs to a higher energetic level, and this shift was observed for all the *Mmuc* strains tested (Figure 7 and Figure 8). *Mmuc* accelerated OXPHOS to enter a metabolic energetic state and consequently increased the basal respiration rate, maximum respiration, and spare respiratory capacity of the macrophages (Figure 7C,D,G). However, we also showed that *Mmuc* infection upregulated proton leakage and that the ATP production levels did not significantly differ between the control and infection groups (Figure 7E,F). We also showed that *Mmuc*-R infection significantly increased the nonmitochondrial oxygen consumption rate, unlike *Mmuc*-ATCC and *Mmuc*-S (Figure 7B). The nonmitochondrial oxygen consumption rate increases in the presence of reactive oxygen species (ROS) and reactive nitrogen species (RNS), and mitochondria are damaged due to the deleterious effects of these reactive intermediates [24,25]. Given these results, *Mmuc*-R more negatively affects the bioenergetic health of macrophages than *Mmuc*-ATCC or *Mmuc*-S. Upregulation of the aerobic glycolysis response is considered a hallmark of proinflammatory signals in both myeloid and lymphoid cells [25]. We showed that infection with all *Mmuc* strains increased glycolysis in infected macrophages, as evidenced by the induced glycolysis and glycolysis capacity rate (Figure 8B,C). In particular, *Mmuc*-R infection significantly increased both glycolysis and the glycolytic capacity rate, unlike the S-type *Mmuc* strains. However, we also showed that *Mmuc* infection reduced the glycolytic reserve rate and the glycolytic reserve as percentage parameters (Figure 8D,F). The glycolytic reserve rate is an important bioenergy source that is activated in response to increases in both glycolysis and the glycolysis capacity rate. Therefore, the reduction in the glycolytic reserve may limit macrophage energy supply when BMDMs need ATP, especially after *Mmuc*-R infection.

## 4. Materials and Methods

### 4.1. Bacterial Culture

The *Mmuc* reference strain (ATCC49650) was purchased from American Type Culture Collection (ATCC, Manassas, VA, USA). The two clinical strains (KMRC 00136-76002 and KMRC 00136-76003) were obtained from The Korean Mycobacteria Resource Center (KMRC) of the Korean Institute of Tuberculosis (Osong, Korea). *Mmuc* strains were grown in Middlebrook 7H9 medium (Difco Laboratories, Detroit, MI, USA) supplemented with 10% oleic albumin dextrose catalase (OADC; BD Biosciences, San Diego, CA, USA) and 0.5% glycerol at 37 °C for 7 days. The number of colony-forming units (CFU)/mL was determined on 7H10 agar supplemented with OADC at 37 °C.

### 4.2. Generation of Bone Marrow-Derived Macrophages

Mouse bone marrow-derived macrophages (BMDMs) were isolated from the femurs and tibias of 7-week-old female C57BL/6 mice (DBL, Chungcheongbuk-do, Korea) [26]. The cells were washed with Dulbecco’s modified Eagle’s medium (DMEM; Biowest, France; L0103-500) and then centrifuged at 440× *g* for 3 min. The pellet was resuspended in 40 mL of DMEM containing glutamine, 1% antibiotic–antimycotics (Biowest, France; L0010-100), 10% fetal bovine serum (FBS; Welgene Co.; Daegu, Korea, S001-01), and 10 ng/mL recombinant mouse M-CSF (JW CreaGene). BMDMs were differentiated in complete medium for a total of 6 days at 37 °C in the presence of 5% CO_2_. On day 3, the media were added, and the culture was maintained for an additional 3 days. After 6 days, nonadherent cells were removed, and the differentiated macrophages were detached by adding trypsin-0.25% EDTA (Biowest, France; L0931-100) for 5 min in an incubator. The detached cells were centrifuged at 440× *g* for 3 min, resuspended, and seeded in complete DMEM.

### 4.3. Growth of M. mucogenicum in BMDMs

BMDMs were plated at a density of 1.5 × 10^5^ cells per 48-well plate. After 24 h, BMDMs were infected at a multiplicity of infection (MOI) of 10 for 4 h at 37 °C. After 4 h, the cells were washed with PBS to remove all extracellular bacteria and further cultivated in fresh complete medium for 5 days. At days 0, 1, 2, 3, 4, and 5, the culture supernatants were aspirated, and cells were lysed by 0.05% Triton X-100 (Sigma, St. Louis, MO, USA). Then, the lysates were plated in tenfold serial dilutions onto 7H10 agar (Becton Dickinson, Franklin Lakes, NJ, USA) to quantify the number of viable bacteria. Colonies were counted after 4 days of incubation at 37 °C. The resultant values were reported as the mean log10 CFU ± standard deviation (SD) per 1.5 × 10^5^ cells.

To determine whether *Mmuc* exhibits similar infection kinetics during the first 4 h, BMDMs were infected at an MOI of 10 per 5 × 10^5^ cells for 30, 60, 120, 180, and 240 min at 37 °C. After incubation, the cells were washed 2–3 times with PBS prewarmed serum-free media to remove extracellular bacteria. The cells were lysed with distilled water containing 0.05% Triton X-100, and the lysates were plated in tenfold serial dilutions onto 7H10 agar to quantify the number of infected bacteria. Colonies were counted after 4 days of incubation at 37 °C. The resultant values are reported as the mean log10 CFU ± standard deviation (SD) per 1.5 × 10^5^ cells.

### 4.4. Scanning Electron Microscopy and Thin-Layer Chromatography (TLC)

Scanning electron microscopy was performed as described with slight modifications [27]. Briefly, 5 mL culture aliquots were concentrated by centrifugation at 1644× *g* for 5 min, and the supernatant was discarded. Then, 1 mL of 4% paraformaldehyde was added to the concentrated cells, and the samples were incubated at room temperature for 2 h. After centrifugation, the concentrated cells were washed twice with 0.05 M sodium cacodylate buffer (pH 7.4). The samples were dehydrated with a sequential ethanol series for 10 min each (50%, 70%, 95%, and 100%). These samples were mixed well with hexamethyldisilazane, a volatile solution, and then placed on the grid before drying. The samples were coated using gold sputter and imaged with a SEM3500M scanning electron microscope.

Bacterial total lipids and GPLs were purified, solubilized, and confirmed as described previously [13]. Total lipids were extracted from *Mmuc* with a chloroform/methanol mixture (2:1, *v*/*v*) by ultrasonication for 20 min and phase-separated by centrifugation. GPLs were purified from total lipid extracts by acetone precipitation. The purified lipids were separated by TLC (Millipore, Billerica, MA, USA) in chloroform/methanol (9:1, *v*/*v*) and detected by spraying with 10% H_2_SO_4_ and heating at 200 °C for 10 min.

### 4.5. Flow Cytometric Analysis

To determine the type of cell death followed by *Mmuc* infection, BMDMs were infected with the bacteria at an MOI of 10. After 24 h, the infected cells were centrifuged at 440× *g* for 3 min, and the supernatant was discarded. Next, the cells were detached by treatment with trypsin for 10 min, and then, 300 µL of complete DMEM was added to each well. Then, we performed experiments according to the manufacturer’s protocol (BD Biosciences, San Jose, CA, USA). Briefly, cells were harvested, washed with ice-cold PBS to remove extracellular bacteria, and then resuspended in 300 µL of 1× binding buffer. Next, 1 × 10^6^ cells were stained with 3 µL of Annexin V-FITC and 3 µL of propidium iodide (PI). Then, these samples were incubated at room temperature for 15 min. After 15 min, 400 µL of binding buffer was added to each tube, and the samples were analyzed using a Cytoflex flow cytometer (Beckman Coulter, Villepinte, France). The cells were assigned to one of four states: alive, annexin V-negative, and PI-negative; early apoptotic, annexin V-positive, and PI-negative; late apoptotic, annexin V-positive, and PI-positive; or necrotic, annexin V-negative, and PI-positive.

### 4.6. Measurement of Cytokines by Enzyme-Linked Immunosorbent Assay (ELISA)

Culture media from the bacteria-infected BMDMs (1.5 × 10^5^ cells/48-well plate) and noninfected BMDMs were collected at 24 h post-infection. Supernatants were collected after centrifugation at 848× *g* for 5 min. The levels of tumor necrosis factor (TNF)-α, interleukin (IL)-6, IL-12p40, and IL-10 were analyzed using commercial ELISA kits and OptEIA ELISA kits (BD Biosciences, San Diego, CA, USA) according to the manufacturers’ instructions.

### 4.7. Western Blot Analysis

After infection with bacteria, adherent cells were washed twice with PBS and then lysed in ice-cold PRO-PREP™ Protein Extraction Solution (Intron, Gyeonggi-do, Korea). After incubation for 10 min, the samples were gently scraped from dishes and then centrifuged at 15,928× *g* for 5 min. The supernatant was collected and stored at −80 °C. The protein concentrations of the lysates were determined using the Pierce BCA Protein Assay kit (Thermo Scientific, Waltham, MA, USA). The protein was mixed with 5× SDS–PAGE loading buffer (LPS solution) and denatured by heating to 100 °C for 15 min. Ten to 30 μg of protein was subjected to electrophoresis on 10~15% polyacrylamide gels containing SDS under reducing conditions. Separated proteins were electroblotted onto 0.22 µm polyvinylidene difluoride (PVDF) membranes (BD), and blots were blocked with 5% skim milk (wt/vol) for 1 h and then washed three times with Tris-buffered saline containing 0.1% Tween 20 (TBS/T). Then, the membranes were incubated overnight at 4 °C with the following antibodies: rabbit anti-p-p38 MAPK (#4631), rabbit anti-p-ERK1/2 (#9101S), rabbit anti-p-JNK (#9251, 1:1000; Cell signaling technology, Boston, MA, USA), and mouse anti-β-actin (#A1978, 1:5000; Sigma-Aldrich, Burlington, MA, USA). Antibody binding was detected using the appropriate secondary antibody coupled with HRP, as described by the manufacturer. Enhanced chemiluminescence was used to detect relevant proteins using the EZ-Western LumiFemto Kit (DG-WF100).

### 4.8. Seahorse Extracellular Flux Analysis

The oxygen consumption rate (OCR) and extracellular acidification rate (ECAR) were measured using a Seahorse XFp Metabolic Flux Analyzer (Seahorse Bioscience, North Billerica, MA, USA). BMDMs were seeded in Seahorse XFp cell culture plates at a density of 80,000 cells per well and cultured at 37 °C in a 5% CO_2_ incubator overnight. The next day, the BMDMs were infected with *Mmuc* for 24 h at an MOI of 10. On the day of analysis, glycolysis stress and mito stress tests were performed according to the manufacturers’ protocols. For the mito stress test, the medium was replaced with XF DMEM supplemented with 10 mM glucose, 1 mM pyruvate, and 2 mM glutamine (pH 7.4) followed by incubation at 37 °C in a non-CO_2_ incubator for 45 min. Oligomycin, carbonyl cyanide phospho-(p)-trifluoromethoxy phenylhydrazone (FCCP), and rotenone/antimycin A were subsequently injected into the medium at final concentrations of 1 μM, 2 μM, and 0.5 μM, respectively. For the glycolysis stress test, the medium was replaced with XF DMEM supplemented with 2 mM glutamine (pH 7.4) followed by incubation at 37 °C in a non-CO_2_ incubator for 45 min. Glucose, oligomycin, and 2-deoxyglucose (2-DG) were subsequently injected into the medium at final concentrations of 10 mM, 1 μM, and 50 μM, respectively. The OCR and ECAR were automatically recorded and calculated by Seahorse XFp software. At the end of the analysis, the medium was removed, and BMDMs were washed with PBS and lysed in RIPA buffer. The protein content in BMDM lysates was measured by the Bradford assay and used for normalization. Data were derived from two independent experiments. Determinants of respiratory and acidification parameters were calculated using the following equation (Supplemental Table S1).

## 5. Conclusions

In conclusion, these results suggest that R-type *Mmuc* is more virulent and induces a stronger immune response than S-type *Mmuc*, similar to other NTM species, such as *M. avium* complex and *M. abscessus* complex. Additionally, R-type *Mmuc* infection negatively affects the bioenergetic condition of macrophages more extensively than infection by the S-type *Mmuc*. This study is meaningful, as this is the first report of differential immune responses to *Mmuc*. These results might also provide a fundamental basis for further studies on the pathogenesis of *Mmuc* as well as appropriate information regarding antibiotic administration and treatment choices that are important for the prognosis of patients.

## Data Availability

Any data or material that support the findings of this study can be made available by the corresponding author upon request.

## Supplementary Materials

The following supporting information can be downloaded at: https://www.mdpi.com/article/10.3390/ijms23052488/s1.
