# Supplementary material for: Comparison of Macrophage Immune Responses and Metabolic Reprogramming in Smooth and Rough Variant Infections of Mycobacterium mucogenicum
Source: Int J Mol Sci. 2022 Feb 24;23(5):2488. doi: 10.3390/ijms23052488 (PMC8910348; doi:10.3390/ijms23052488)
Supplement: Supplementary file 1 [file ijms-23-02488-s001.zip › ijms-1578784-SI.pdf]

Supplemental table S1.

Parameter Value	Equation
Non-mitochondrial oxygen consumption	Minimum rate measurement after R/A injection
Basal respiration	(Last rate measurement before first injection) - (Non-mitochondrial respiration rate)
Maximum respiration	(Maximum rate measurement after FCCP injection) - (Non-mitochondrial respiration rate)
H^+(proton) leak	(Minimum rate measurement after Oli injection) - (Non-mitochondrial respiration rate)
ATP production	(Last rate measurement before first injection) - (Minimum rate measurement after Oli injection)
Spare respiratory capacity	(Maximal respiration) - (Basal Respiration)
Glycolysis	(Maximum rate measurement before Oli injection)- (Last rate measurement before Glu injection)
Glycolytic capacity	(Maximum rate measurement after Oli injection) - (Last rate measurement before Glu injection)
Glycolytic reserve	(Glycolytic capacity) - (Glycolysis)
Non-glycolytic acidification	Last rate measurement prior to glucose injection
Glycolytic reserve as a %	(Glycolytic capacity rate) / (Glycolysis) x 100
